# Radial extracorporeal shock wave therapy: a novel approach for the treatment of Dupuytren's contractures: A case report

**DOI:** 10.1097/MD.0000000000020587

**Published:** 2020-06-12

**Authors:** Stefano Brunelli, Cinzia Bonanni, Marco Traballesi, Calogero Foti

**Affiliations:** a Fondazione Santa Lucia, Scientific Institute for Research, Hospitalization and Health Care; b Physical and Rehabilitation Medicine, Tor Vergata University of Rome, Rome, Italy.

**Keywords:** acquired hand deformities, patient relevant outcome, range of motion, rehabilitation

## Abstract

**Introduction:**

While the efficacy of focused Extracorporeal Shock Wave Therapy (ESWT) in the treatment of Dupuytren's disease (DD) is supported by one positive trial, the effects of radial ESWT is unclear.

**Patient concerns:**

A 79-year-old man with a 4-year history of impairment of left-hand function and pain due to DD with weakness and flexion deformities of middle and ring fingers. He has not been treated before for this impairment.

**Diagnosis:**

The diagnosis of DD was based on clinical features and ultrasound images.

**Interventions:**

Four weekly sessions of radial ESWT with 1400 impulses 3 bar each, 12 Hz.

**Outcomes:**

The limitations in activities of daily living were analyzed through the Disabilities of Arm Shoulder and Hand Questionnaire (DASH) and Michigan Hand Outcome Questionnaire (MHQ) at baseline, after four sessions of radial ESWT and at 4-months follow-up. Data analysis showed a significant reduction of hand deformities and an improvement of daily living performance. The effects continued at the 4-months follow-up.

**Conclusion:**

This case report demonstrates the feasibility of radial ESWT. Radial ESWT sessions may be carried out by a physiotherapist in outpatient clinics with cost reduction compared with surgical treatment and focused ESWT. Radial ESWT is a non-invasive, well tolerated therapy, so it should be considered in the DD treatment.

## 1 Introduction

Dupuytren's disease (DD) is a progressive disease due to thickening of palmar fascia that generates cords and nodules and it implies loss of range of motion of the affected fingers and pain with tenosynovitis.^[1]^ DD commonly affects male individuals, over 50 years of age with various risk factors (alcoholism, smoking, thyroid problems, liver disease, diabetes, previous hand trauma, epilepsy, possibly due to anti-convulsive medication) and with certain genetic predisposing factors (autosomal dominant with variable penetrance). DD is characterized by fibrotic stage that ensues from fibrocellular proliferation. The ring finger followed by the little and middle fingers are most commonly affected.

Diagnosis is usually based on signs and symptoms. Patients, initially, might be symptomless and they might present retraction of palmar fascia associated with nodules, at a later stage their ability to hold objects and pain progressively decrease.

Surgery is often the main treatment in progressive DD with selective or total aponeurectomy.^[2]^ Alternative non-surgical treatments are enzymatic fasciotomy^[3]^ with collagenase injections^[3]^ and percutaneous needle fasciotomy.^[4]^ Even radiotherapy was reported to successfully prevent disease's progression in early stage of Dupuytren's contracture.^[5]^

Extracorporeal shock wave therapy (ESWT) is an effective and safe non-invasive treatment option for many orthopedics diseases and its efficacy is clearly supported by positive outcome compared to placebo or alternative treatments as reported by Schmitz et al,^[6]^ but this recent review does not mention the efficacy of ESWT on DD.

ESWT exerted a positive overall effect on pain and function for lower-limb tendinopathy^[7]^ and given the progressive fibroproliferative nature of DD as calcific shoulder tendinopathy or Peyronie's disease, in which ESWT was successfully applicable, it was hypothesized similar effects on DD in reducing contracture and pain.^[8]^ Shock wave is a mechanical vibration, an acoustic wave which promotes, through mechano-transduction, the tissues’ regeneration.^[9]^ Radial ESWT (rESWT), compared with conventional focused ESWT, is a low- to medium-energy shock wave generated when a projectile is accelerated by compressed air and hits an applicator. Radial shock wave devices develop their maximum energy at the probe tip and distribute it radially into the tissue, while focused shock waves converge deeply to a target area. Radial ESWT has broader and safer effect than focused shock wave therapy.^[10]^ Considering that DD contractures are superficial, we hypothesized that a treatment with rESWT was effective to reduce them and improve hand function.

## 2 Case report

**Figure 1:**
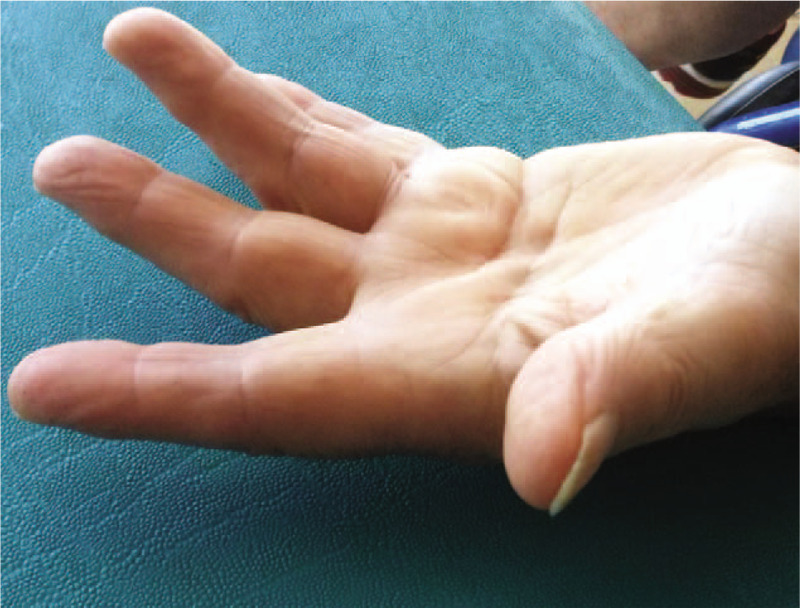
Clinical evaluation before treatment: thickening of palmar fascia and flexion deformities of middle and ring fingers with a total flexion deformity of 30°.

A 79-year-old man, retired concierge, affected by diabetes, hypertension and prostatic hypertrophy, presented with a 4-year history of impairment of left-hand function, pain and difficulty grasping and opening a jar due to hand weakness and flexion deformities of middle and ring fingers. He has not been evaluated neither treated before for this impairment. The pain started to get worse recently. On physical examination, on palpation, he presented thickening of the palmar skin, nodules and cords, and table top test positive. On the basis of clinical features and ultrasound images, we diagnosed DD (Fig. 1).

We used a goniometer to measure the degree of finger's contraction and to determine the Tubiana grading system. Tubiana grading system assigns a score to the extension deficit of a digit by summing the total deficit angles of the metacarpophalangeal joint proximal interphalangeal and distal interphalangeal joints. The Tubiana stages are: N = palmar nodule without presence of contracture; 1 = total flexion deformity between 0° and 45°; 2 = between 45° and 90°; 3 = between 90° and 135°; 4 = >135°.^[11]^

We analyzed the daily limitation of DD in activities of daily living through the Disabilities of Arm Shoulder and Hand Questionnaire (DASH) and Michigan Hand Outcome Questionnaire (MHQ). Nowadays there are no Dupuytren-specific outcome questionnaires validated in the literature. DASH has 30 questions. Its domains are specific daily functions, symptoms (pain, strength, and sensation), impact on life of the patient and general disability and handicap. There are no questions investigating satisfaction. The minimal score is 30, the maximum is 150. The responses are summed to form a raw score that is then converted to a 0-to-100 scale with a lower score indicating higher degree of autonomy.^[11]^ We have excluded two items from DASH score: writing (the patient was right-handed) and sexual activity (widower), but the overall score is not affected because up to 3 missing answers are allowed.^[12]^

The MHQ was devised from the University of Michigan in 1998. It has 37 items and measures six domains: overall hand function, activities of daily living, work performance, pain, aesthetics, and patient satisfaction with hand function. Scores on MHQ range from 0 to 100, with a lower score indicating higher degree of disability.^[13]^

Even if the DASH and MHQ are both specific measures of upper extremity function, the DASH addresses global upper-extremity disability and symptoms, while MHQ includes aesthetic and satisfaction aspects differently than DASH, and for these reasons we administered both questionnaires.

The patient underwent 4 weekly sessions of rESWT (device: Shock Med SW1352, EME srl, Italy) with 1400 impulses 3 bar each, 12 Hz. We used a concave head with 9 mm diameter; each treatment lasted 2 min. We treated the area in which there was the palmar retraction interposing ultrasound gel and using a continuous movement along the tendons, associated with a 10 seconds of single point application in correspondence of nodules and cords. No orthotic device was utilized throughout the study period. The participant provided written consent to participate voluntarily in this clinical case report. Personal information was made anonymous. Our patient presented a total flexion deformity between 0° and 45° (30°), stage 1 by Tubiana. The DASH and MHQ results at first evaluation were 32.1 and 54, respectively (Fig. 1).

The worst limitations the patient reported were “Use a knife to cut food” and “Recreational activities” and even severe hand pain “in any specific activity.” These difficulties limited social activity of the patient that used to visit friends for dinner and card-playing. The patient reported a temporary worsening of symptomatology for 24 h after the first 2 single sessions, but the overall hand function was improved (DASH score: 22.3). No adverse effects were reported.

**Figure 2:**
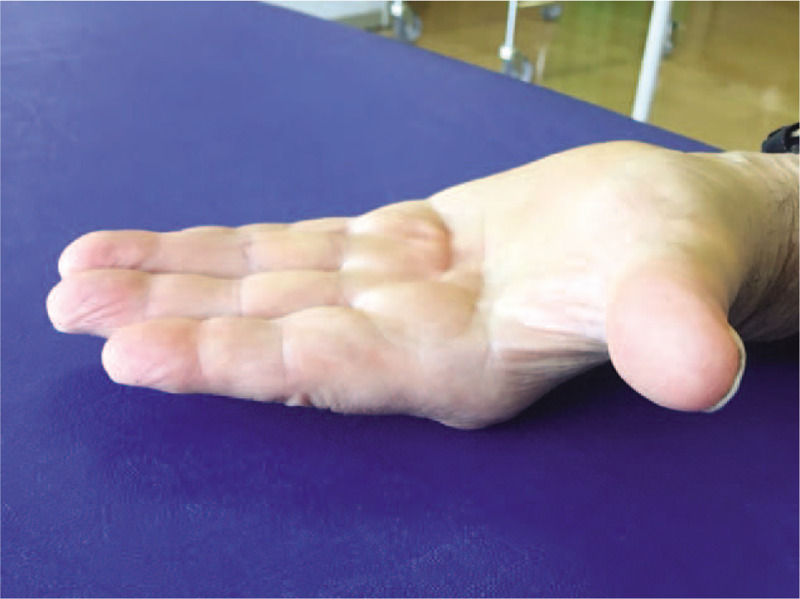
One week after the fourth session of radial ESWT. Reduction of nodules and cord dimensions in the palmar fascia with a total flexion deformity of 15°.

One week after the fourth session of rESWT, we observed a reduction of nodule and cord's dimension in the palmar fascia of left-hand. The total flexion deformity was reduced to 15° (stage 1 of Tubiana) (Fig. 2).

He described no difficulty in grasping and using small objects. He was very happy because he went back to getting out of the house and to see his friends (he reported “mild difficulty” and “mild pain” in the above-mentioned questions). He presented an improvement in both scales (score 8.9 in DASH and 76 in MHQ).

At the 4-month follow-up, the patient had maintained a similar hand function (score 10.7 in DASH and 75 in MHQ). Finally, we observed a reduction of flexion deformity of the affected fingers.

Tubiana's grading does not improve because this classification presents an ample range for each stage, anyways the patient showed a reduction of 15° total flexion deformity, with functional gain and increase of self-confidence. Table 1 summarizes all the assessments made throughout the study.

**Table 1:**
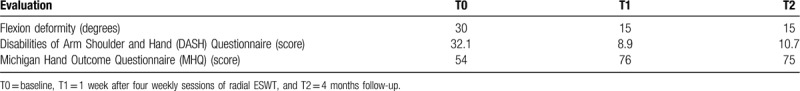
Scores and degrees assessments throughout the study.

As showed by Franchignoni's research report, the smallest improvement in DASH score to reflect a change that is clinically meaningful for the patient is 10.81 points.^[14]^ With this premise, we observe an important patient's response to rESWT, with an improvement of 23.2 points at the end of treatment. These beneficial effects continue also at the 4-month follow-up, with DASH improvement of 21.4 points and score 75 in MHQ.

## 3 Discussion

Extracorporeal shock wave therapy is a safe non-invasive treatment option and it might be a tool for prevention of the progression and for treatment of DD. Surgery treatment is not free from complications and these operations undertaken in early stages of DD is considered to be associated with a higher rate of recurrence.^[2]^

Enzymatic fasciotomy with collagenase injections seems to be effective for reducing the contractures, but it requires an expert surgeon and it is not free from complications (skin rupture, tendon rupture, hematoma, pain, lymphangitis, and also severe allergic reactions or anaphylactic shock).^[3]^ Collagenase injection and needle fasciotomy have 3-year recurrence rates of about 45% each.^[4]^

Radiotherapy is based on X-ray, so this would be a possible risk about development of complications in the radiated region. One of the biological effects of ESWT is the removal of damaged matrix constituents.^[15]^ Moreover, in an MRI study after ESWT treatment, the authors have found that the improvement in clinical symptoms of fasciitis plantaris is due to the action of stimulation of soft tissue healing by removal of inflammatory debris, as we have speculated in DD.^[9]^ The treatment with rESWT is well tolerated and anesthesia is not necessary.

The efficacy of ESWT in the treatment of DD is supported by positive outcome reported by Notarnicola et al.^[16]^ However, in this trial they used focused shock waves with in-line ultrasound guidance (Minilith SL1, Storz, Switzerland) in 3 sessions at 5 days intervals and each treatment consisted in less impulses than our case report (1000) with less frequency (4 Hz) and intensity (0.03 mJ/mm^2^): these differences are presumably due to the different device feature. In the study of Notarnicola et al, the average DASH score of all patients at first evaluation was similar (mean score 37.0) to our patient. In our case report, the improvement of 23.2 in DASH score is a minimal clinically important difference (10.8) for the patient,^[3]^ while in the cited study the mean improvement seems not to reach the minimal clinically important difference.^[16]^

The Professional Practice Committee of the Physical and Rehabilitation Medicine Section of the Union of European Medical Specialists suggests the use of ESWT in DD as it may decrease the need for surgery.^[17]^

We want to emphasize that a low intensity rESWT session may be carried out by physiotherapists in outpatient clinics with cost reduction compared with surgical treatment and focused ESWT. High-intensity focused ESWT needs ultrasound or radiographic guide and the equipment is much more expensive.

## 4 Conclusion

Radial ESWT is a non-invasive, cheap and well-tolerated therapy. It seems to be effective in the DD treatment. Further studies, including randomized controlled trials, are needed to evaluate the efficacy of radial ESWT in DD and its comparable effects to focused ESWT.

## Acknowledgments

Thanks to Livia Brunelli, Durham University, UK, for the English language editing.

## Author contributions

**Conceptualization:** Stefano Brunelli.

**Data curation:** Cinzia Bonanni, Stefano Brunelli.

**Investigation:** Cinzia Bonanni, Stefano Brunelli.

**Supervision:** Calogero Foti, Marco Traballesi.

**Visualization:** Stefano Brunelli.

**Writing – original draft:** Cinzia Bonanni.

**Writing – review & editing:** Stefano Brunelli, Calogero Foti, Marco Traballesi.
